# Angiotensin II Activates Yes-Associated Protein (YAP) in Fibroblast Promoting Deep Fascia Remodeling

**DOI:** 10.3390/ijms262211105

**Published:** 2025-11-17

**Authors:** Brasilina Caroccia, Ilaria Caputo, Giovanni Bertoldi, Valentina Favaro, Andrea Angelini, Andrea Benetti, Lucia Petrelli, Piero Di Battista, Maria Piazza, Pietro Ruggieri, Raffaele De Caro, Carla Stecco, Carmelo Pirri

**Affiliations:** 1 Department of Medicine-DIMED, University of Padova, 35128 Padova, Italy; ilaria.caputo@unipd.it (I.C.); giovanni.bertoldi@unipd.it (G.B.); valentina.favaro.11@studenti.unipd.it (V.F.); andrea.benetti@unipd.it (A.B.); maria.piazza@unipd.it (M.P.); 2 Department of Orthopedics and Orthopedic Oncology, University of Padova, 35128 Padova, Italy; andrea.angelini@unipd.it (A.A.); pietro.ruggieri@unipd.it (P.R.); 3 Department of Neurosciences, Institute of Human Anatomy, University of Padova, 35121 Padova, Italy; lucia.petrelli@unipd.it (L.P.); pierodibattista.phd@gmail.com (P.D.B.); raffaele.decaro@unipd.it (R.D.C.); carla.stecco@unipd.it (C.S.)

**Keywords:** fascia, thoracolumbar fascia, angiotensin II, Ang II type 1 receptor, yes associated protein (YAP), fibroblasts, remodelling

## Abstract

The deep fascia, traditionally regarded as a passive structural tissue, is now recognized as a metabolically and biologically active structure where biochemical signals and biomechanical forces interact to influence proprioception, pain, force transmission, and adaptation to mechanical load. In this study, the convergence point between Angiotensin II (Ang II) signaling via its receptor, Angiotensin type 1 receptor (AT1R), and the mechanosensor Yes-associated protein (YAP) was investigated in human fascial fibroblasts. The presence of angiotensin II (Ang II) receptors was confirmed in fibroblasts from the deep fascia, with the AT1 receptor being the most prevalent subtype. Short-term exposure to Ang II (15–30 min) caused YAP dephosphorylation and its translocation to the nucleus, indicating YAP activation. Notably, prolonged Ang II treatment (7 days) significantly increased the expression of fibrosis-related genes, including collagen types I and III (*COL1A1*, *COL3A1*), and hyaluronan binding protein 2 (*HABP2*). This gene expression was decreased by pretreatment with the AT1R antagonist irbesartan or the YAP inhibitor verteporfin. Additionally, Ang II promoted fibroblast proliferation/migration, key features of fibrotic progression, through AT1R-dependent pathways. These findings show that Ang II acts as both a biochemical and biomechanical signal in the deep fascia, activating YAP signaling and promoting fibrotic remodeling. Our results uncover a new Ang II–YAP pathway in fascial fibroblasts, offering potential targets for therapy in fibrosis and related conditions involving the deep fascia.

## 1. Introduction

Fasciae form a vast network of connective tissue that surrounds, separates, and connects muscles, bones, nerves, and blood vessels, supporting the structural integrity and coordinated function of the human body. Among the various fasciae, the deep fascia has traditionally been considered a passive mechanical structure, mainly serving as a containment layer for muscles and soft tissues. However, over the past twenty years, increasing evidence has challenged this traditional view, demonstrating that deep fascia is a metabolically and biologically active tissue involved in proprioception, pain modulation, force transmission, and adapting dynamically to mechanical stimuli [1,2].

Recent studies have further demonstrated that fibroblasts in connective tissues maintain extracellular matrix (ECM) homeostasis through Yes-associated protein and transcriptional coactivator with PDZ-binding motif (YAP/TAZ) signaling, highlighting the dynamic response of connective tissue to both biochemical and mechanical cues [3,4].

The thoracolumbar fascia (TLF) serves as a model for understanding the complex functions of deep fasciae. Anatomically, the TLF is an aponeurotic structure that is essential for the biomechanical stability of the trunk and for coordinating segmental forces between the spine, pelvis, and lower limbs [5]. Histologically, the TLF is defined by a dense collagenous extracellular matrix (ECM) composed of collagen types I and III (COL1A1 and COL3A1), elastin fibers, glycosaminoglycans, and embedded fibroblasts, which together influence its mechanical properties and functional versatility [1,2,6].

Notably, deep fascia is a target for systemic hormonal regulation, such as sex hormones [7] or the renin-angiotensin system (RAS) [8]. Traditionally, regarded as an endocrine regulator of blood pressure and electrolyte homeostasis, the RAS is now recognized to exert local, paracrine, and autocrine effects on tissue remodeling and fibrosis [9,10].

The primary effector of the RAS, Angiotensin II (Ang II), mediates its biological effects through two main G-protein-coupled receptor (GPCR) subtypes: the Ang II type 1 receptor (AT1R) and the Ang II type 2 receptor (AT2R) [10,11]. Activation of AT1R is linked to pro-fibrotic, pro-inflammatory, and hypertrophic effects, while AT2R and the Mas receptor (MasR), activated by Ang 1-7, are involved in counter-regulatory, anti-fibrotic responses [12]. Beyond its traditional role in the cardiovascular system, irbesartan, an AT1R receptor antagonist, has been shown to decrease fibrotic remodeling in myocardial tissue, [13] highlighting the therapeutic significance of RAS inhibition in connective tissue disorders. Although the role of the RAS in organ fibrosis in the heart, kidney, liver, and skin [7,11] has been extensively studied, its potential involvement in fascial remodeling remains unexplored. In a pioneering study, Pirri et al. investigated the expression of RAS components in human TLF samples obtained from patients undergoing elective spinal surgery [8]. *AT1R* was the most abundantly expressed receptor subtype, with detectable levels of *AT2R* and *MasR*, while the enzymes responsible for local Ang II production—angiotensin-converting enzyme 1 and angiotensin-converting enzyme 2—were minimally expressed or undetectable [8]. These findings indicate that the deep fascia lacks an autonomous local RAS and instead responds to circulating Ang II, likely originating from systemic sources. The high levels of AT1R expression suggest that the fascia could be susceptible to Ang II-driven pro-fibrotic signaling, similar to mechanisms observed in other fibrotic diseases [14]. Furthermore, the relative expression of counter-regulatory receptors (AT2R and MasR) indicates the presence of an intrinsic, although limited, modulatory system within the fascia that helps balance the pro-fibrotic effects of AT1R activation [8].

Furthermore, the deep fascia is increasingly recognized as an important site of mechanotransduction, the process by which cells detect and respond to mechanical stimuli through intracellular signaling pathways. Among the mechanotransducers identified in various connective tissues, the Yes-associated protein (YAP) has become a crucial regulator of fibroblast behavior and ECM remodeling [15,16,17]. YAP is a transcriptional co-regulator whose activity is tightly controlled by cytoskeletal tension, extracellular matrix stiffness, and mechanical deformation. When activated, YAP moves to the nucleus, where it influences the expression of genes involved in cell proliferation, matrix deposition, and fibrogenesis [18,19]. In musculoskeletal disorders, for example, abnormal activation of YAP/TAZ has been linked to persistent fibroblast activation and matrix deposition. At the same time, broader studies confirm its essential role in the pathobiology of bone, cartilage, and muscle [20].

Recent work by Pirri et al. provided the first direct evidence of YAP expression and activation in human TLF fibroblasts [21]. Using an experimental model with focal extracorporeal shockwaves (fESWs) to mimic mechanical stimulation, the authors demonstrated a significant increase in the nuclear localization of YAP, along with higher expression of *COL1A1* and Hyaluronan Binding Protein 2 (*HABP2*), two genes involved in ECM composition and fascial stiffness. Pharmacological inhibition of YAP with verteporfin blocked these responses, confirming the crucial role of YAP in regulating mechano-responsive gene expression in fascial fibroblasts [21]. These findings emphasize YAP as a critical mediator of fascial tissue response to mechanical stress, with significant implications for both normal remodeling processes and pathological fibrosis. The convergence of mechanical and hormonal stimuli in regulating fascial homeostasis and remodeling prompts intriguing questions about their potential interaction and mutual reinforcement. Ang II significantly influences the activation of YAP, a crucial component of the Hippo signaling pathway, particularly in the context of fibroblast function and fibrotic disease progression.

Research shows that Ang II can inactivate the Hippo pathway by impairing the activity of the large tumor suppressor kinase (LATS), thereby increasing nuclear translocation of YAP. In cardiac fibroblasts, this mechanism promotes proliferation and transdifferentiation into myofibroblasts, contributing to cardiac fibrosis. Specifically, Ang II-mediated YAP activation has been shown to increase the expression of fibrotic markers, such as collagen type I [14,22]. Recent evidence suggests that pharmacological inhibition of YAP-TEAD signaling in vitro reduces pulmonary fibrosis [23]. Additionally, YAP/TAZ is increasingly recognized as a mechanosensitive transducer that links ECM stiffness to bone and connective tissue disorders [24]. Moreover, research indicates that blocking YAP activation can reduce Ang II-induced fibrotic responses. For example, inhibition of YAP in fibroblasts reduces myocardial fibrosis and improves cardiac function after Ang II stimulation [19,25]. Similarly, in renal tissues, suppression of YAP activation has been associated with decreased fibrosis and renal injury in hypertensive models [26,27].

These findings highlight the key role of Ang II in regulating YAP activity, suggesting a potential therapeutic target for the treatment of fibrotic diseases.

Furthermore, both YAP-mediated mechanotransduction and Ang II/AT1R signaling share common downstream effectors, including TGF-β, the migration pathway, and cytoskeletal reorganization, all of which are involved in fibroblast activation, ECM deposition, and tissue stiffening [16,17,28]. Mechanical loading and circulating hormonal signals may work together to promote fascial adaptation under normal conditions or contribute to pathological fibrosis in response to prolonged or dysregulated stimuli.

Therefore, this study aims to improve our understanding of the molecular interactions between mechanotransducers and hormonal signaling within the human deep fascia. We examined the role of Ang II and its receptor, AT1R, in activating YAP using deep fascia fibroblasts as an in vitro model. This process results in increased ECM deposition, as well as fibroblast migration and proliferation, thereby contributing to fibrosis and tissue remodeling.

## 2. Results

### 2.1. AT1R, AT2R, and MasR Expression in Deep Fascia Fibroblasts

**Figure 1:**
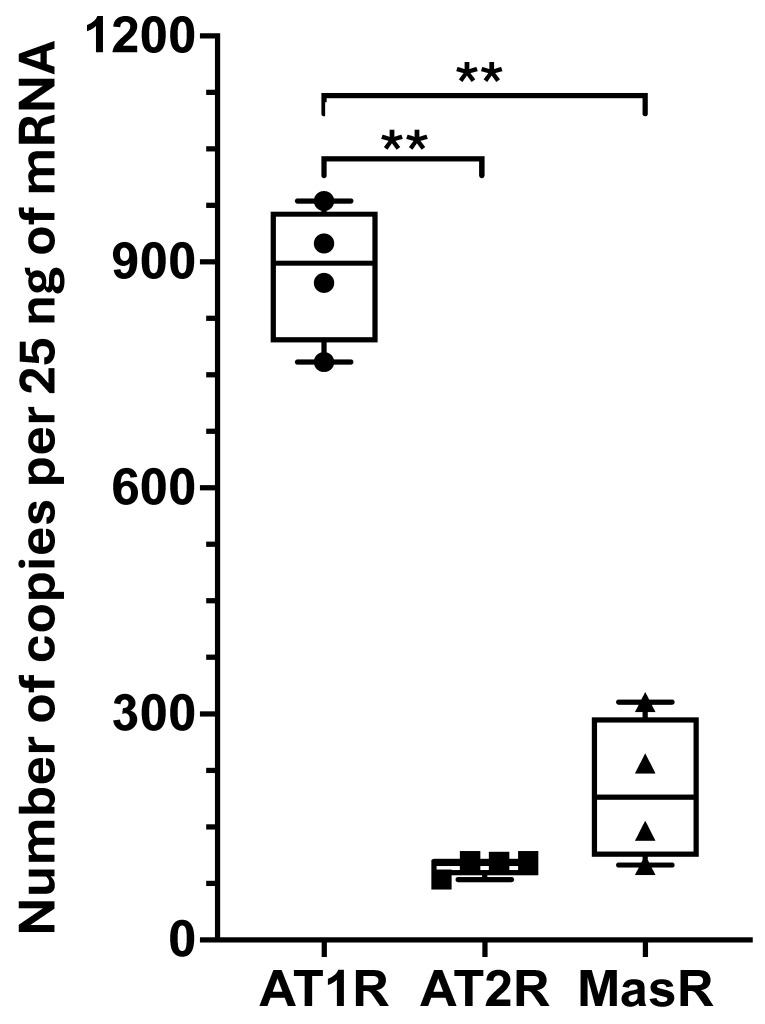
Gene expression of angiotensin receptors in human fibroblasts from TLF was analyzed. The number of gene copies of angiotensin receptors (*AT1R*, *AT2R*, *MasR*) in TLF was measured using ddPCR and normalized to the total amount of mRNA used in the reaction. *AT1R* was the most highly expressed receptor, followed by *MasR* and then *AT2R.* Data are presented as gene copies from 25 ng per mRNA. **: *p* < 0.001.

Fibroblasts were obtained from TLF tissue samples of four patients who underwent elective spine surgeries, as previously reported [21]. The expression of angiotensin receptors, such as *AT1R*, *AT2R*, and *MasR*, was measured in fibroblasts using ddPCR. We observed high levels of *AT1R* and lower levels of *MasR* and *AT2R* (Figure 1). The receptor expression ranking in fibroblasts, *AT1R* > *MasR* > *AT2R*, was consistent with that observed in TLF tissue [8].

### 2.2. Angiotensin II Promotes YAP Activation

**Figure 2:**
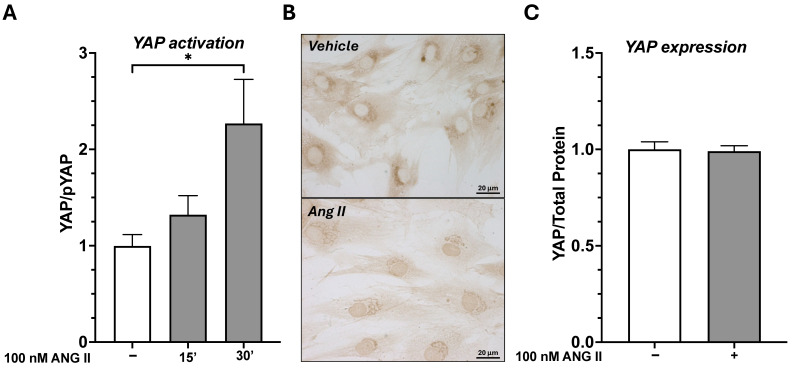
Ang II modulates YAP activation but not its expression. (**A**) Automated protein analysis shows an increased YAP/pYAP ratio in fibroblasts treated with 100 nM Ang II for 15 and 30 min compared to untreated cells. All data represent the mean ± SD of 4 experiments, each performed in duplicate. * *p* < 0.01. (**B**) Immunohistochemistry images show YAP nuclear translocation upon Ang II treatment, whereas in control cells, YAP is mostly localized in the perinuclear area. (**C**) Automated protein analysis revealed that Ang II did not alter the total expression of YAP after 7 days of treatment (mean ± SD of 4 experiments, each performed in duplicate).

To determine whether YAP can be activated in response to Ang II, fibroblasts from TLF were seeded in 6-well plates and treated with 100 nM Ang II for 15 and 30 min. After treatment, proteins were extracted, and both total and phosphorylated YAP levels were analyzed by immunoblotting. We observed that the ratio of active to inactive YAP (YAP/p-YAP) began to increase at 15 min and was significantly higher in fascial fibroblasts exposed to 100 nM Ang II than in untreated cells after 30 min (Figure 2A). This finding was consistent with strong YAP nuclear immunolabeling observed after fibroblasts were exposed to Ang II, as shown in Figure 2B. In contrast, in untreated cells, the YAP immunosignal was observed in the perinuclear region (Figure 2B). This suggests that Ang II promotes YAP dephosphorylation and its nuclear translocation, where YAP can activate gene expression by binding to DNA. We also investigated whether Ang II increased YAP levels in fibroblasts after 7 days of treatment. Ang II was added to fibroblasts every 24 h, and after 7 days, proteins were extracted to measure YAP levels. Our results showed that although Ang II influences YAP activation, it does not alter its expression levels over time (Figure 2C).

### 2.3. Ang II Increases the Expression of Collagen Type 1 A (COL1A1), Collagen Type 3 A (COL3A1), and Hyaluronan Binding Protein 2 (HABP2)

We exposed fibroblasts to Ang II for 7 days to investigate whether Ang II could induce the expression of genes associated with fibrosis. We measured the gene expression of *COL1A1* and *COL3A1* after treating the cells with 100 nM Ang II for 7 days. Ang II significantly increased *COL1A1* and *COL3A1* gene expression at day 7 (Figure 2A,B; 8 independent experiments, each performed in triplicate). To clarify whether AT1R mediated this effect, cells were treated with Ang II and the AT1R antagonist irbesartan. Pretreating fascial fibroblasts with 10 μM irbesartan prevented the Ang II-induced increase in gene expression of *COL1A1* and *COL3A1* (Figure 2A,B; 8 independent experiments, each performed in triplicate).

**Figure 3:**
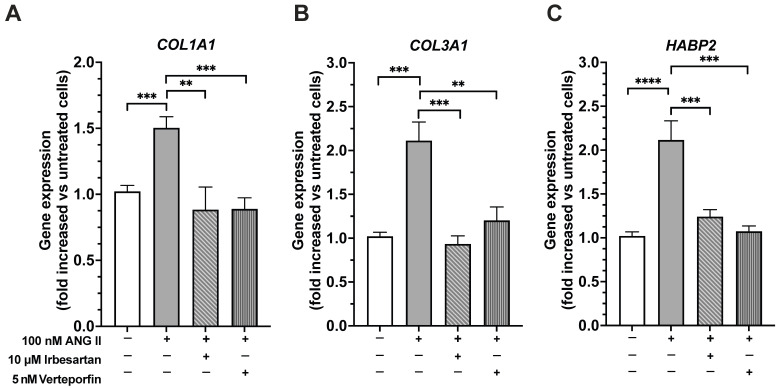
Ang II treatment enhanced *COL1A1* (**A**), *COL3A1* (**B**), and *HABP2* (**C**) gene expression at day 7. Treatment with irbesartan, an AT1R antagonist, or with verteporfin, a YAP inhibitor, prevented the effects of Ang II. Gene expressions were evaluated by real-time PCR. All data represent mean ± SD of 8 experiments, each performed in triplicate. ** *p* < 0.001; *** *p* < 0.0001, **** *p* < 0.00001.

To clarify whether Ang II promotes the expression of fibrosis-related genes through YAP activation, we treated fibroblasts with 100 nM Ang II on top of 5 nM verteporfin, a YAP inhibitor. Verteporfin significantly decreased the Ang II-induced upregulation of the genes *COL1A1* and *COL3A1* (Figure 3A,B; 8 independent experiments, each in triplicate).

We also evaluated hyaluronan-binding protein 2 (*HABP2*) gene expression, a marker of fascial remodeling, after Ang II treatment alone or in combination with irbesartan or verteporfin. Our results showed that after 7 days of exposure, Ang II significantly increased *HABP2* gene expression (Figure 3C; 8 independent experiments, each performed in triplicate). Pretreatment of fibroblasts with irbesartan reduced the Ang II-induced upregulation of *HABP2* (Figure 3C; 8 independent experiments, each performed in triplicate). The same results occurred when YAP activity was blocked with 5 μM verteporfin.

Notably, experiments were also conducted with 10 μM irbesartan and 5 nM verteporfin alone to assess non-specific effects of these drugs on gene expression in fibroblasts. We found that irbesartan alone and verteporfin alone did not alter the expression of the gene of interest.

### 2.4. Ang II Promotes Cell Proliferation/Migration in Fibroblasts

**Figure 4:**
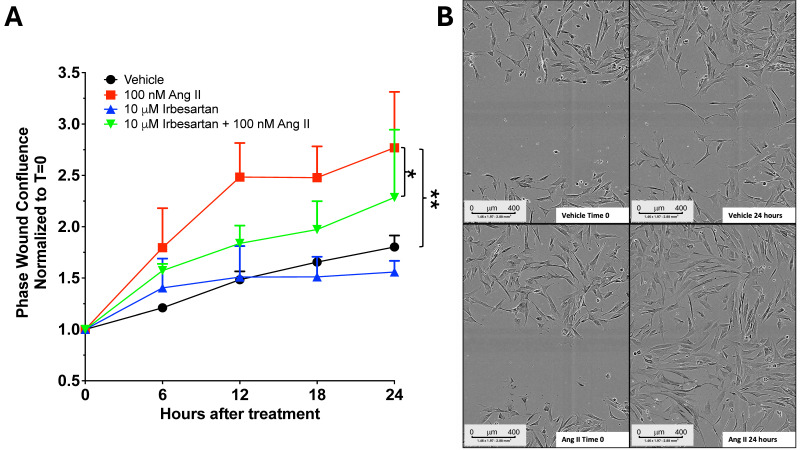
Ang II induced fibroblast cell proliferation/migration through AT1R activation (**A**). This process was assessed using a live-cell wound healing assay. Fibroblasts from four patients (two women, two men) were tested in sextuplicate. After 5 days of stimulation with 100 nM Ang II, either alone or combined with 10 μM irbesartan, cells were plated at 10,000 per well in an ImageLock 96-well plate (Sartorius). A uniform scratch was created using the Incucyte^®^ 96-Well WoundMaker Tool, and wound closure was continuously monitored with the Incucyte S3 Live-Cell Analysis System (Sartorius). Images were taken every 3 hours over a 24-h period using a 10× objective. The extent of wound closure by migrating and dividing cells was quantified over time with Incucyte analysis software. Data from each well were normalized to the initial time point, averaged per donor, and combined across all four patients. Statistical significance was assessed using two-way repeated-measures ANOVA (factors: treatment and time), followed by Tukey’s test for multiple comparisons. Results are expressed as mean ± SEM from four donors, each with six replicates, * *p* = 0.03; ** *p* = 0.006. Representative images display wound closure at 0 and 24 h, with or without Ang II (**B**).

We performed a wound healing assay to assess whether Ang II could stimulate fibroblast proliferation/migration. Fibroblasts were treated for 7 days with either Ang II alone or with Ang II combined with the AT1R antagonist irbesartan. On day 7, a wound was created, and the movement of fibroblasts was monitored every 6 h for 24 h. After 7 days of Ang II treatment, fibroblasts displayed a proliferative/migratory phenotype, indicated by an increased cell index (*p* < 0.001 in Ang II-treated cells compared to untreated; Figure 4A,B; 4 independent experiments, each in sextuplicate). Irbesartan inhibited cell proliferation/migration, suggesting these processes are mediated through the AT1R receptor (Figure 4A). In Figure 4B, images show wounded cell monolayers at 0 and 24 h after wounding, with or without Ang II.

### 2.5. Gelatin-Degrading Activities of MMP-2 and MMP-9

**Figure 5:**
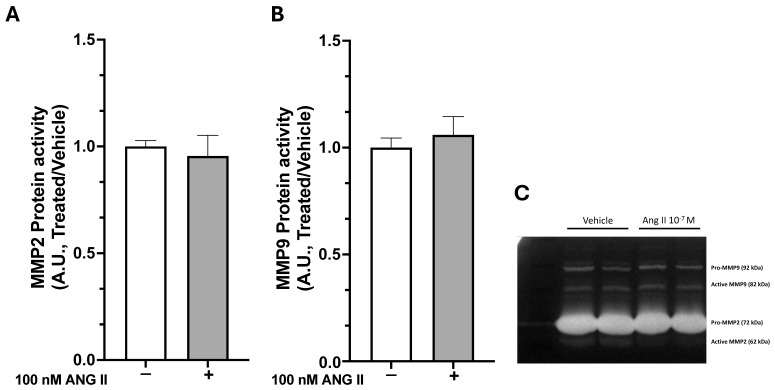
MMP-2 and MMP-9 activity were measured in deep fascia fibroblasts after 7 days of treatment. The bar graphs show that Ang II treatment did not affect the activity of MMP-2 (**A**) or MMP-9 (**B**). (**C**) displays a zymography gel with bands indicating gelatinolytic activity in the medium from both untreated cells and Ang II-treated cells. All data represent mean ± SD of 5 experiments, each performed in duplicate.

The balance between collagen synthesis and breakdown is crucial for ECM deposition and fibrosis. We assessed MMP-2 and MMP-9 activity in cell culture media after 7 days of treatment with 100 nM Ang II. The gelatin-degrading activities of MMP-2 and MMP-9 did not change significantly after Ang II treatment (Figure 5A–C), indicating that Ang II promotes ECM accumulation.

### 2.6. Transcriptomic Changes in Ang II-Treated Fibroblast

**Figure 6:**
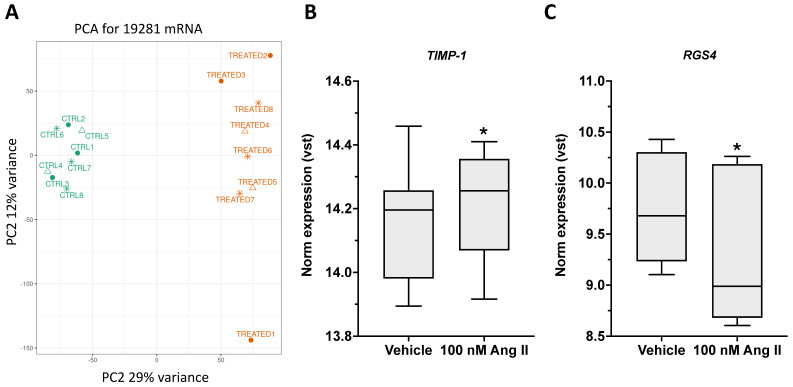
Principal Component Analysis (PCA) revealed distinct clusters for the Ang II-treated (orange) and untreated fibroblasts (green) (**A**). Expression levels of *TIMP-1* (Tissue Inhibitor of Metalloproteinases-1) (**B**) and *RGS4* (Regulator of G-protein Signaling 4) (**C**) were obtained through RNAseq in deep fascia fibroblasts after 7 days of treatment with 100 nM Ang II (* *p* < 0.05).

We performed RNA-seq analysis on deep fascia fibroblasts treated with Ang II for 7 days to detect changes in gene expression and identify key pathways activated by Ang II. Principal Component Analysis (PCA) revealed distinct clusters for the treated and untreated groups, highlighting the convergence of biological replicates (Figure 6A). We identified 19,281 genes; among these, a total of 389 differentially expressed genes (DEGs) were identified, with 153 upregulated and 236 downregulated (adj *p*-value < 0.05). Enrichment analyses based on the Reactome pathways database, revealed that these DEGs were mainly related to Extracellular Matrix Organization, Collagen Biosynthesis and Modifying Enzymes, and Collagen Formation (Table 1). The Log2 fold changes of DEGs between treated and untreated samples ranged from −0.67 to 0.48 and included *COL15A1*, *COL16A1*, *COL5A1*, *COL3A1*, and *COL1A2*.

Notably, *TIMP-1* (Tissue Inhibitor of Metalloproteinases-1), a key regulator of ECM remodeling, was up-regulated (Log2FC: 0.09) (Figure 6B). TIMP-1 inhibits the activity of MMPs, including MMP-9, MMP-1, and MMP-3, thereby preventing the breakdown of ECM components such as collagen, elastin, fibronectin, and laminin [29]. This inhibition reduces collagen degradation and limits ECM breakdown, promoting ECM accumulation, fibrosis, and tissue stiffening. Notably, the most significantly down-regulated gene was *RGS4* (Log2FC: −0.67), also known as Regulator of G-protein Signaling 4, which acts as a brake on GPCR signaling (Figure 6C) [30]. RGS4 inhibits Angiotensin II–AT1 receptor signaling by accelerating GTP hydrolysis in Gα_q/11_ and Gα_i/o_ proteins [30]. The observed reduction in *RGS4* expression, driven by Ang II itself, prolongs G-protein signaling and enhances downstream pathways, such as collagen deposition. This may contribute to the development of sustained fibrosis and tissue remodeling.

## 3. Discussion

Cells respond to chemical stimuli by activating specific receptors that trigger intracellular biochemical signaling pathways. In addition to chemical cues, mechanical stimuli can trigger biochemical responses via mechanosensors, specialized cellular components that detect mechanical stimuli and convert them into biochemical signals. This process enables cells to respond to changes in their physical environment. These sensors include ion channels, cell adhesion molecules, and cytoskeletal elements. Furthermore, nuclear components, such as the transcriptional regulators YAP/TAZ, function as mechanotransducers, integrating mechanical inputs to regulate gene expression [31]. Together, these diverse mechanosensors coordinate complex cellular behaviors, such as migration, proliferation, and differentiation, in response to mechanical cues [23]. Although GPCRs are mainly known for their chemosensory roles, growing evidence indicates they also act as mechanosensors [31]. GPCRs can be indirectly activated through paracrine signaling, in which mechanical forces trigger the release of chemical mediators, but they can also function as direct mechanosensors at the cell membrane. Several studies support the idea of direct mechanical activation of GPCRs, although the exact molecular mechanisms remain to be clarified [32]. Notably, the GPCR AT1R is also recognized as a ‘mechanical sensor’ that converts mechanical stress into intracellular biochemical signals [33].

YAP, a key effector of the Hippo signaling pathway, has emerged as a crucial mediator of cellular responses to both mechanical and biochemical stimuli [15]. Ang II, a potent vasoactive peptide involved in blood pressure regulation and the pathophysiology of cardiovascular diseases, has been shown to influence YAP activity. Ang II promotes YAP nuclear translocation and transcriptional activity, mainly by inducing cytoskeletal reorganization and inhibiting the upstream kinases LATS1/2, which phosphorylate YAP and retain it in the cytoplasm [22]. This activation enhances the expression of YAP target genes, which are involved in cell growth, fibrosis, and inflammation. In vascular smooth muscle cells and cardiac fibroblasts, the Ang II–YAP axis plays a role in vascular remodeling and cardiac hypertrophy. YAP may also regulate components of the renin-angiotensin system, indicating a two-way interaction that intensifies harmful signaling in hypertensive and fibrotic conditions [22].

In recent years, Pirri et al. have made significant contributions to the field of fascia mechanotransduction [8]: they reported the expression of RAS components in deep fascia and described the role of YAP in response to mechanical stimuli [21]. The detection of angiotensin receptors (*AT1R*, *AT2R*, and *MasR*) in fascial tissues suggests that Ang II may play a role in fascial remodeling, fibrosis, and inflammation, similar to its effects in other connective tissues like the myocardium, kidneys, and skin [8,9,14]. These findings shed light on the dual function of fascia as both a structural and hormonally responsive tissue, offering new insights for exploring endocrine-related fascial disorders and treatment approaches. In the present study, the expression of Ang II receptors in fibroblasts obtained from deep fascia was investigated, and *AT1R* was found to be the most abundantly expressed subtype. Using fibroblasts as an in vitro model, the role of Ang II in promoting abnormal ECM remodeling and facilitating fascial fibrosis through YAP activation was studied. Upon Ang II stimulation, dephosphorylation of YAP and its translocation to the nucleus after 30 min of treatment were observed. Ang II affected YAP activity without altering its overall expression levels, as prolonged exposure to Ang II (7 days) did not change the total amount of YAP. Notably, 7 days of Ang II treatment significantly increased the expression of genes associated with extracellular matrix organization, collagen biosynthesis and modifying enzymes, and collagen formation, enhancing fibroblast proliferation/migration through AT1R signaling. This profibrotic effect was prevented by pretreatment with irbesartan, an AT1R antagonist. Additionally, Ang II significantly increased *HABP2* gene expression, a marker of fascial remodeling, through AT1R activation. Fibroblast proliferation and migration is essential in both normal tissue repair and the development of fibrosis. Ongoing fibroblast activation and migration lead to excess ECM deposition and increased tissue stiffness, which can impair organ function. Fibroblasts respond to various signals, including growth factors, cytokines, and changes in the ECM. These ECM changes, such as higher collagen levels and increased *TIMP-1* expression, which blocks MMP enzymatic activity, further impact fibroblast activity, creating a self-perpetuating pro-fibrotic cycle.

This study redefines fascial fibrosis as a druggable, signal-driven disorder by identifying a key convergence node, the Ang II/AT1R-YAP module, that jointly controls fascial extracellular matrix remodeling and its transcriptional reprogramming. Unlike previous research that examined collagen buildup or cellular movement as separate processes, we used fascia-derived fibroblasts and longitudinal, multimodal tests to delineate the connection between receptor activation at the cell membrane and downstream intracellular pathways. We showed that clinically approved AT1R antagonists (e.g., irbesartan) and YAP inhibitors (e.g., verteporfin) can block each step of this harmful cascade [21]. This identifies the Ang II/AT1R-YAP axis as a dual-precision therapeutic target: upstream inhibition benefits from drug classes with well-established cardiovascular safety profiles, while downstream modulation provides transcriptional specificity within fascial tissues.

The present study advances and expands the existing body of work in several ways. In previous investigations, we focused on characterizing the molecular expression profile of RAS components in human thoracolumbar fascia and on documenting the presence of YAP signaling in fascial fibroblasts in response to mechanical stimuli. These studies were descriptive, establishing the foundational evidence that deep fascia is not a passive structure but instead a hormonally responsive and mechanosensitive tissue [8,21]. In contrast, the current research moves beyond descriptive characterization and directly examines the functional crosstalk between RAS activation and YAP signaling. We demonstrated that Ang II, acting through the AT1R, can induce YAP nuclear translocation and upregulate profibrotic genes in deep fascia fibroblasts. By combining pharmacological inhibition with functional assessments of ECM deposition, our study established a mechanistic link between systemic hormonal cues and intracellular mechano-responsive pathways.

Furthermore, this study identified YAP as a crucial point where hormonal and mechanical signals converge. Such an approach has not been explored in the scientific literature. Conceptually, this work shifts the focus of deep-fascia research from a late-stage, matrix-focused perspective to an early-stage, signal-interception paradigm, making it suitable for prophylactic or regenerative-focused interventions. Although this evidence was collected in an in vitro model, this study supports repurposing antihypertensives for fascial indications, accelerates clinical translation by leveraging established safety profiles, and initiates a pipeline of next-generation, YAP-biased ligands tailored to fascial fibro-pathologies. Collectively, these advances reveal not only a previously unrecognized mechanistic axis but also provide a strategic blueprint for signal-guided, fascia-targeted anti-fibrotic therapies poised to transform the management of myofascial disorders.

### Limitations and Future Perspectives

Although this study provides new evidence for the role of Ang II in regulating YAP activation and promoting profibrotic responses in deep fascia fibroblasts, some limitations must be acknowledged. The lack of in vivo validation is a major gap, as fibrosis involves complex interactions among fibroblasts, immune and endothelial cells, ECM remodeling, and systemic factors. Future research should employ animal models, such as Ang II-infused mice or experimental models of fascial fibrosis, to verify histological collagen buildup, YAP nuclear localization, and changes in tissue biomechanics. Additional studies should also examine the timing and reversibility of this pathway, determining whether brief YAP inhibition can reduce established fibrosis or if its role is limited to the early phase of fibroblast activation. Lastly, expanding this research to include outcomes beyond structural changes, such as stiffness, pain, and functional impairment, will be essential to translating these mechanistic insights into effective antifibrotic treatments of fascia.

## 4. Materials and Methods

### 4.1. Cell Isolation and Treatment

Fibroblasts were isolated from TLF of 8 patients (4 women and 4 men) and cultured, as previously detailed [21]. Fresh tissues were rinsed in phosphate-buffered saline with antibiotics, minced into small pieces, and cultured in DMEM/F-12 medium (Thermo Fisher Scientific, Milan, Italy) supplemented with 10% fetal bovine serum and 1% penicillin–streptomycin (Sigma-Aldrich, Milan, Italy) at 37 °C in 5% CO_2_. After about a week, fibroblasts migrated from the tissue explants. Residual tissue fragments were then removed, and the cells were detached using trypsinization, centrifuged, and reseeded in fresh culture medium, which was renewed every 3 days [21]. To evaluate the effect of Ang II, fibroblasts were seeded in 6-well plates at 50,000 cells/well or in 12-well plates at 20,000 cells/well, for protein and gene expression, respectively. To measure *COL1A1*, *COL3A1,* and *HABP2* gene expression, and YAP total amount, cells were treated for 7 days with Angiotensin II 100 nM (Cod. #A9525, Sigma-Aldrich, Milan, Italy) alone or on top of 10 µM of irbesartan, the angiotensin Type 1 receptor antagonist (Cod. #I2286, Sigma-Aldrich, Milan, Italy). Irbesartan was added to fresh media 30 min before 100 nM Ang II. Fresh stimuli were added every 24 h. The concentrations of 100 nM Angiotensin II and 10 µM irbesartan were chosen based on previous studies demonstrating strong activation of fibroblast signaling pathways [14]. Experiments were also performed in the presence of 5 nM verteporfin (Cod. #SML0534, Sigma-Aldrich, Milan, Italy), an inhibitor of YAP activity. To determine the optimal concentration of verteporfin for 7-day experiments, we performed a dose–response analysis, using cell viability as the endpoint, given that YAP activity is essential for cell survival. Concentrations ranging from 5 μM to 1 nM were tested, and 5 nM was identified as the optimal concentration that did not affect cell viability during prolonged exposure. To assess the short-term effect of Ang II on YAP phosphorylation, cells were seeded in 6-well plates at 100,000 cells per well, grown to approximately 80% confluence, and stimulated with 100 nM of Ang II for 15 or 30 min.

### 4.2. RNA Extraction and Real Time PCR

Total RNA was extracted from fibroblasts with the Single Cell RNA Purification Kit (Cod. # NR51800, Sial, Rome, Italy) following manufacturer’s protocol. 500 ng total RNA was then reverse-transcribed with SensiFAST cDNA Synthesis Kit, (Cod. # BIO-65054, Aurogene, Rome, Italy) in a final volume of 20 µL. Then, 100 ng cDNA were used to perform real-time RT-PCR using 4X CAPITAL qPCR Green Master Mix (Cod. #BR0501702, Biotechrabbit, Berlin, Germany). PCR was performed using the CFX96™ instrument (Bio-Rad Laboratories, Milan, Italy) with the following protocol: 95 °C for 3 min, followed by 40 cycles of 95 °C for 15 s, and 60 °C for 30 s. The relative expression levels of *COL1A1*, *COL3A1* and *HABP2* mRNA were measured with real-time RT-PCR and calculated using the comparative Ct (2^−ΔΔCt^) method, using porphobilinogen deaminase, *PBGD,* as a housekeeping gene. Primers used are reported in Table 2.

### 4.3. Protein Expression

Total YAP and phosphoYAP protein levels were evaluated using JESS Simple Western™ instrument (ProteinSimple^®^, Bio-Techne, Abigdon, UK). Briefly, fibroblasts were sonicated in RIPA lysis and extraction buffer containing protease inhibitors (Cod. #C0001, Protease Inhibitor Cocktail, TargetMol, Boston, MA, USA) and phosphatase inhibitors (Cod #C0002, Phosphatase Inhibitor Cocktail I, TargetMol, Boston, MA, USA), and the protein concentration was determined with BCA (Cod. #23225, (Thermo Fisher Scientific, Milan, Italy)). Samples were diluted to total protein concentrations of 1 μg/μL in 0.1× sample buffer and 1× fluorescent master mix (EZ standard pack I; Bio-Techne, Abigdon, UK) and 3 µL were added per well following manufacturer instructions. The polyclonal rabbit IgG anti-YAP (cod. #4912, Cell Signalling, Milan, Italy), anti-phosphoYAP (cod. #4911, Cell Signalling, Milan, Italy), were diluted 1:50 into milk free Antibody diluent. The secondary Ab (anti-rabbit HRP) and enhanced chemiluminescence (ECL) reagents were used according to the kit’s instructions (anti-rabbit detection module chemiluminescence, #DM-001; Bio-Techne, Abigdon, UK). We run RePlex assay (Re-Plex module #RP-001, Biotechne, Abigdon, UK) to remove primary and secondary antibodies and run Total Protein assay (Total Protein Detection module #DM-TP01, Bio-Techne, Abigdon, UK) in each single run. Therefore, data were normalized by total protein content as automatically provided by the instrument.

### 4.4. YAP Immunostaining

Fibroblasts were seeded on coverslips at a density of 20,000 cells/slide and cultured for 48 h. Cells were treated with 100 nM Ang II for 30 min. After treatment fibroblasts were fixed with 4% paraformaldehyde for 15 min, and were then permeabilized with 0.3% Triton X-100 (Cod # 93443, Sigma-Aldrich, Milan, Italy) in PBS for 10 min. The monoclonal antibody YAP (Cod #12395, Cell Signalling, Milan, Italy) diluted in 0.1% Triton PBS, was applied to the cells and incubated at 4 °C overnight. After 3 washes with PBS, cells were then incubated with a goat anti-mouse HRP-conjugate antibody (Cod #P026002 Agilent Dako, Santa Clara, CA, USA) at room temperature for 1 h. Positive immunostaining was detected with 3,3′–diaminobenzidine (Cod # K346711-2 Liquid DAB; Agilent Dako, Santa Clara, CA, USA); the reaction was developed for 1 min and stopped with water. Observations were carried out using a DM 2000 (Leica, Wetzlar, Germany) microscope. Negative control sections were prepared omitting the use of a primary antibody.

### 4.5. Wound Healing Assay

After 5 days of stimulation with 100 nM Ang II alone or combined with 10 μM irbesartan, fibroblasts were seeded at a density of 10,000 cells per well in an ImageLock 96-Well Plate (Sartorius, Surrey, UK) in sextuplicate, continuing stimulation until day 7. Afterwards, a scratch was made using the Incucyte^®^ 96-Well Woundmaker Tool (Sartorius, Surrey, UK), a device designed to create uniform, 700–800 µm-wide wounds in each well simultaneously. Following two PBS washes, fresh cell media with or without stimuli were added to each well, and the plate was placed in the Incucyte S3 Live-Cell Analysis System (Sartorius, Surrey, UK) to monitor cell migration and proliferation over time. Each well was scanned every 3 h for 24 h, generating time-lapse microscopy images with a 10× objective (Incucyte S3 Live-Cell Analysis System, Sartorius, Surrey, UK). Data were analyzed with Incucyte image software to determine the percentage of wound area occupied by cells over time. Using an adherent cell-by-cell analysis, cell density values (cells/mm^2^) were obtained from each image (mean ± SEM). The data from each well were pooled and averaged across four patients used in these experiments, and then normalized to time zero.

### 4.6. Gelatin Zymography

To evaluate metalloprotease activity, cell culture media were collected after treatment with 100 nM Ang II. Equal amounts of secreted proteins (10 μg) were mixed with 2x Laemmli Sample Buffer (Bio-Rad Laboratories, Milan, Italy) and loaded onto 8% acrylamide gels containing 1% gelatin (Sigma-Aldrich, St. Louis, MO, USA) as substrates for MMP-9 and MMP-2. Electrophoresis was performed in a running buffer (192 mM glycine, 25 mM Tris base, 0.1% SDS) at 120 V for 2 h. Afterwards, gels were washed twice with 2.5% Triton X-100 for 30 min each, then incubated overnight at 37 °C in developing buffer (50 mM Tris-based, 200 mM NaCl, 5 mM CaCl_2_, pH 7.4). The following day, gels were stained with 0.5% (*w*/*v*) Coomassie Brilliant Blue R-250 (Sigma-Aldrich, St. Louis, MO, USA) in a solution of 40% methanol and 10% acetic acid, then destained in the same solution. Gelatinases appear as clear bands against the blue background. Images were acquired using ATOM UVITEC (Uvitec, Milan, Italy), and the relative enzyme amounts were quantified by measuring band intensity with the Nine Alliance Program (Uvitec, Milan, Italy).

### 4.7. RNA Sequencing Analysis in Ang II-Treated Fibroblasts

RNA sequencing was performed on primary fibroblasts treated with 100 nM Ang II for 7 days. Total RNA was extracted using the Single Cell RNA Purification Kit (Cod. # NR51800, Sial, Rome, Italy) and quantified with the Qubit broad range RNA assay (Thermo Fisher Scientific, Milan, Italy) on a Qubit^®^ 4.0 Fluorimeter, following the manufacturer’s protocol. RNA integrity was evaluated using RNA 6000 Nano kit with Agilent via the Agilent Bioanalyzer 2100 (Agilent Technologies Italia S.p.A., Milan, Italy). For library preparation, 100 ng of total RNA was used with the Illumina^®^ Stranded mRNA Prep, Ligation (16 Samples) kit (Illumina Italy S.r.l., Milan, Italy) following the manufacturer’s instructions. The quality and quantity of NGS libraries were assessed using the Sensitivity DNA kit on the Agilent Bioanalyzer 2100, resulting in an average fragment size of approximately 280 bp across all samples, and by a Qubit dsDNA HS Assay (Thermo Fisher Scientific, Milan, Italy) on a Qubit^®^ 4.0 Fluorometer, according to the manufacturer’s instructions. Equal molar amounts of each sample were combined to form the final pool, which was then diluted and denatured to reach a final library concentration of 1.7 pM. The pooled library, supplemented with 1% PhiX DNA control (Illumina Italy S.r.l., Milan, Italy), was sequenced using the NextSeq 500/550 High Output Kit v2.5 (150 cycles) with a 75 bp paired-end protocol on a NextSeq™ 550 System (Illumina, San Diego, CA, USA). Reads in paired-end fastq files were analyzed using an in-house Python (v.3.12.3) pipeline: in the workflow reads were tested for quality control with FastQC (v.0.12.1) [34], then adapter sequences, bad quality and duplicated reads were removed by Fastp (v.0.23.4) (average quality > 25, read length < 40) [35]. Next, processed reads were mapped using STAR (v.2.7.10b) [36] on the GRCh38 human genome assembly and quantified with RSEM (v.1.3.1) [37].

The statistical analysis was performed using packages and custom scripts in an R environment (v.4.5.1): tables and plots were assembled using dplyr (v.1.1.4) and ggplot2 (v.3.5.2) [38]. The expression matrix with raw counts of identified mRNAs was normalized and assessed for differential mRNAs expression with DESeq2 (v.1.48.2) [39], considering Benjamini–Hochberg adjusted *p*-values ≤ 0.05 as significant threshold.

### 4.8. Statistical Analysis

Statistical analyses were performed using GraphPad Prism version 10 (GraphPad Software Inc., San Diego, CA, USA). A *p*-value < 0.05 was considered statistically significant. To improve clarity, two levels of significance were reported: *p* < 0.05 and *p* < 0.01. Most comparisons achieved *p* < 0.01, while some parameters displayed *p*-values between 0.01 and 0.05. Data distribution was tested with the Kolmogorov–Smirnov test to confirm normality. Quantitative data are presented as mean ± standard deviation (SD) unless otherwise specified. Differences in YAP activation (active versus inactive forms) and gene expression between treated and untreated cells were analyzed using unpaired *t*-tests or Mann–Whitney tests when normality was not assumed. Fibroblast proliferation and migration curves were analyzed with two-way repeated-measures ANOVA (factors: treatment and time), followed by Tukey’s multiple comparison test. For time-course experiments, results are reported as mean ± standard error of the mean (SEM).

## 5. Conclusions 

Our findings reveal a critical role for Ang II in regulating YAP activation and promoting profibrotic responses in deep fascia fibroblasts. We demonstrate that Ang II induces YAP to translocate to the nucleus without altering its overall levels, suggesting post-translational regulation as a key mechanism. This activation increases the expression of ECM-related genes, suggesting a pathway through which Ang II contributes to fascial remodeling and fibrosis. Importantly, these effects are mediated via the AT1R receptor, as pharmacological inhibition with irbesartan effectively reduces the fibrotic response. Additionally, Ang II encourages fibroblast migration, further supporting its role in modulating the extracellular matrix. Overall, our data suggest that the Ang II–YAP axis could be a new therapeutic target for preventing or reversing fibrosis in fascia and possibly other connective tissues.

## Figures and Tables

**Table 1:** Pathways up- and down-regulated in Ang II-treated fibroblasts according to enrichment analysis.

Pathway	Overlap	Combined Score	*p*-Value	Adj *p*-Value	Odds Ratio	Regulation	Genes
Collagen Biosynthesis and Modifying Enzymes	12/67	932.472	9.2 × 10^−14^	1.2 × 10^−11^	31.070	UP	*ADAMTS2; COL15A1; COL3A1; COL16A1;* *COL1A2; COL5A1; P4HA2; COL6A2; COL6A1;* *SERPINH1; COL6A3; PCOLCE*
Collagen Formation	12/90	576.364	3.6 × 10^−12^	3 × 10^−10^	21.883	UP	*ADAMTS2; COL15A1; COL3A1; COL16A1; COL1A2;* *COL5A1; P4HA2; COL6A2; COL6A1; SERPINH1;* *COL6A3; PCOLCE*
Extracellular Matrix Organization	22/300	415.430	9.4 × 10^−16^	3.8 × 10^−13^	12.006	UP	*LAMA5; COL15A1; COL16A1; TGFB1; TNC;* *LTBP4; BGN; PCOLCE; FBLN1; NID2; FBLN2;* *ADAMTS2; COL3A1; EFEMP1; COL1A2;* *COL5A1; P4HA2; COL6A2; COL6A1;* *SERPINH1; COL6A3; TIMP1*
Extracellular Matrix Organization	7/300	5.589	6.5 × 10^−2^	1.7 × 10^−1^	2.040	DOWN	*VCAN; ITGA3; CTSK; SPP1; LAMB1; THBS1; ICAM1*

**Table 2:** Primers used for gene expression analysis with real time PCR.

Gene (Accession Number)	Primer Forward	Primer Reverse
*COL1A1* NM_000088.4	5′-TTCTCAGCGTGGGTAAGTGT-3′	3′-TTCTCAGCGTGGGTAAGTGT-5′
*COL3A1* NM_000090.4	5′-CTGGACCCCAGGGTCTTC-3′	5′-GACCATCTGATCCAGGGTTC-3′
*HABP2* NM_001177660.3	5′-AATGGCTCTGGTGGGAAAGA-3′	3′-TTCTCAGCGTGGGTAAGTGT-5′
*PBGD* NM_000190.3	5′-TGCCCTGGAGAAGAATGAAG-3′	3′-AGATGGCTCCGATGGTGA-5′

## Data Availability

The original contributions presented in this study are included in the article. Further inquiries can be directed to the corresponding authors.

## Abbreviations

The following abbreviations are used in this manuscript:

TLF	thoracolumbar fascia
ECM	extracellular matrix
RAS	renin-angiotensin system
Ang II	Angiotensin II
GPCRs	G-protein-coupled receptors
AT1R	Ang II type 1 receptor
AT2R	Ang II type 2 receptor
MasR	Mas receptor
ACE2	angiotensin-converting enzyme 2
YAP	Yes-associated protein
p-YAP	phospho- Yes-associated protein
fESWs	focal extracorporeal shockwaves
COL1A1	Collagen type 1 A
COL3A1	Collagen type 3 A
HABP2	Hyaluronan Binding Protein 2
